# Effect of abdominal binder on shoulder pain after laparoscopic gynecologic surgery: A randomized, controlled trial

**DOI:** 10.1097/MD.0000000000034127

**Published:** 2023-06-23

**Authors:** Yoon Jung Kim, So Yeong Hwang, Hee-Soo Kim

**Affiliations:** a Department of Anesthesiology and Pain Medicine, Seoul National University College of Medicine, Seoul National University Hospital, Seoul, Korea.

**Keywords:** abdominal binder, ambulation time, laparoscopic surgery, pulmonary recruitment maneuver, shoulder pain

## Abstract

To evaluate the impact of abdominal binder (AB) use on postoperative shoulder pain in patients recovering from laparoscopic gynecologic surgery.

**Methods:**

This was a prospective randomized study conducted in a tertiary teaching hospital. Overall, 144 patients underwent laparoscopic gynecologic surgery. The postoperative use of an AB for 24 hours was added to the pulmonary recruitment maneuver.

**Results:**

Of 144 consenting patients, 72 patients each were allocated to the AB and control groups, respectively, and 14 patients were excluded. Finally, 130 patients were analyzed, with 68 in the AB group and 62 in the control group. There was no difference in the incidence of postoperative shoulder pain between the 2 groups (55.9% vs 56.5%, *P* = 1.000). The severity of the worst shoulder pain, measured using a numerical rating scale, did not differ between the 2 groups (Median [interquartile range] 2 [0–5] vs 2 [0–5]; *P* = .865). The severity of surgical site pain, pain and nausea medications, and the ambulation time were not different in the 2 groups.

**Conclusion:**

The use of an AB was not beneficial for postoperative shoulder pain following laparoscopic gynecologic surgery. Surgical site pain, ambulation time, and postoperative nausea and vomiting were not improved with the use of an AB.

## 1. Introduction

Laparoscopic techniques are widely used in gynecological surgeries worldwide. Compared with laparotomy, laparoscopic techniques reduce blood loss, postoperative pain, and hospital stay^[1,2]^; however, postoperative shoulder pain can occur. In laparoscopic gynecologic surgeries, the frequency of postoperative shoulder pain ranges from 35 to 83%.^[3,4]^ Shoulder pain after laparoscopic surgery is frequently positional and can be triggered by deep breathing; thus, shoulder pain could slow down recovery.^[5]^

The exact mechanism of shoulder pain after laparoscopic surgery remains unclear; however, the main hypothesis is that residual carbon dioxide (CO_2_) in the abdominal cavity stimulates the phrenic nerves thus causing shoulder pain.^[6,7]^

A previous study reported that the pulmonary recruitment maneuver in the Trendelenburg position significantly reduced the intensity and frequency of shoulder pain in patients who underwent laparoscopic gynecological surgery.^[3]^ The pulmonary recruitment maneuver could transfer CO_2_ caudal to reduce irritation of phrenic nerve. However, despite the maneuver, 63% of patients reported shoulder pain. In addition to the maneuver, we hypothesized that postoperative use of an abdominal binder (AB) could facilitate the absorption of residual CO_2_ into the peritoneum and pelvis through physical pressure. According to Henry law, the amount of gas dissolved in a liquid is proportional to its partial pressure above the liquid.

This study aimed to evaluate the impact of AB use on postoperative shoulder pain in patients recovering from laparoscopic gynecologic surgery. We assessed the impact of AB use on surgical site pain, ambulation time, and postoperative nausea and vomiting as a secondary aim.

## 2. Methods

The Institutional Review Board of the Seoul National University College of Medicine approved this study (number: H-2107-220-1240, date: August 24, 2021). The study was registered at ClinicalTrials.gov before patient recruitment (NCT05043844, September 13, 2021, principal investigator: Hee-Soo Kim). Only patients who provided written informed consent were enrolled to participate in this study. We conducted this study in compliance with the Good Clinical Practice guidelines and the Helsinki Declaration, and the report was written based on the applicable Consolidated Standards of Reporting Trials guidelines.

### 2.1. Eligibility criteria

This study was conducted at the Seoul National University Hospital. We included adult patients aged 20 to 79 years with American Society of Anesthesiologists Physical Status classification I to III who underwent elective laparoscopic gynecological surgery under general anesthesia.

The exclusion criteria were patients who had conversion to open surgery from laparoscopic surgery, changes in intraabdominal pressure due to surgical difficulties, a previous shoulder disease or history of shoulder surgery, difficulty wearing an AB due to skin disease or wounds in the abdomen, and development of postoperative subcutaneous emphysema. Moreover, we excluded cases, in which we could not evaluate the occurrence of shoulder pain because of early discharge or patient refusal to participate in the survey.

### 2.2. Randomization

Before patient recruitment, a clinical research coordinator who was not involved in this study generated and kept a random allocation sequence table with a one-to-one ratio using the R software (version 4.1.3; R Foundation for Statistical Computing, Vienna, Austria). Patients were allocated to 2 groups: the control (C) and AB groups. Owing to the nature of the AB, concealing the intervention from patients and the anesthetist performing the intervention applying the AB was not possible. However, researchers who collected the data or analyzed the records were separated and blinded.

### 2.3. Protocol

The anesthetic and surgical management of the patients were performed in the same manner, except that an AB was worn after surgery in the AB group. To prevent postoperative nausea and vomiting, 5 mg of dexamethasone and 0.075 mg of palonosetron were injected intravenously before inducing anesthesia. Total intravenous anesthesia was used for the induction and maintenance of anesthesia (target-controlled infusion, 4 mg/mL of 2% propofol, and 4 ng/mL of remifentanil). After loss of consciousness, 0.6 mg/kg rocuronium was administered intravenously to facilitate endotracheal intubation. After the anesthetist confirmed a train-of-four count of 0, endotracheal intubation was performed. The ventilation mode was volume-controlled with the tidal volume set at 8 mL/kg for the ideal body weight. During the maintenance of anesthesia, the end-respiratory CO_2_ concentration was maintained between 30 and 40 cmH_2_O. Intraperitoneal CO_2_ insufflation was maintained at a flow rate of 12 L/min and intraperitoneal pressure at 12 mm Hg. The laparoscopic procedures were performed in the full Trendelenburg position. At 30 minutes before the end of surgery, 30 mg ketorolac and 1 g acetaminophen were administered intravenously for postoperative pain control. When the laparoscopic procedure ended, CO_2_ was passively drained. At the end of surgery, the attending anesthesiologist placed the operating table in the Trendelenburg position at 30° and performed a pulmonary alveolar recruitment maneuver (continuous positive airway pressure 45 cmH_2_O administered 5 times for 7 seconds). The pulmonary recruitment maneuver was performed by one anesthesiologist in charge of this research (Y.J.K.).

In the AB group, an elastic AB was worn by another researcher (S.Y.H.) in the Trendelenburg position (elastic AB, Soosung Medical, Seoul, Korea). Sugammadex was used to reverse muscle relaxation before placing the patient in a supine position. The AB was worn for 24 hours.

Sugammadex was also used to reverse muscle relaxation in the control group, and extubation was performed. The patient was placed in the supine position. The patients were transferred to the post-anesthesia care unit. To control postoperative pain, a multimodal analgesia approach was employed. This included intravenous patient-controlled analgesia, with zaltoprofen administered 3 times a day. Additionally, acetaminophen or fentanyl was administered when the patient experienced pain rated at 4 or above on the numerical rating scale, or when requested by the patient.

### 2.4. Measurement

The incidence of postoperative shoulder pain was the primary outcome measure. The follow-up duration was 36 hours postoperatively. At 12, 24, and 36 hours postoperatively. After transfer to general ward, a trained research nurse visited patients and explained how and when to fill out the pain diary and interviewed pain characteristics during follow-up period. The patients recorded shoulder pain and surgical site pain at the pain diary using numerical rating scale. In addition, it was recorded whether shoulder pain occurred at any time during the follow-up period. If shoulder pain was present, the worst pain score was recorded. Patients were also asked whether the shoulder pain was related to the position. We assessed the frequency and the scores of surgical site pain at 12, 24, and 36 hours postoperatively. Rescue pain and nausea medications, and ambulation time after surgery were recorded.

### 2.5. Statistical analysis

Data are presented as numbers (proportions) for categorical variables, while data for continuous variables are presented as means (standard deviations) or medians (interquartile ranges) depending on the normality of the data distribution evaluated using the Shapiro–Wilk test. We used the chi-square test or Fisher exact test to compare categorical variables and Student *t* test and Mann–Whitney *U* test to compare continuous variables with normal and skewed distributions, respectively. Statistical significance was set at *P* value < .05. To compare pain scores measured 3 times, 2-way repeated measure analysis of variance was conducted, and statistical significance set at *P* value < .0167 after Bonferroni correction. Statistical analyses were performed using the R software.

### 2.6. Sample size calculation

A previous study reported the incidence of postoperative shoulder pain as 63% in patients who underwent laparoscopic gynecologic surgery with pulmonary recruitment maneuver.^[3]^ The sample size was calculated by assuming a 40% reduction in the incidence of postoperative shoulder pain in the experimental group compared to that in the control group. The sample size was 61 patients in each group, using an *α* error of 0.05 and power (1 − β) of 0.8. Considering a 15% dropout rate of participants, we recruited 72 patients for each group. The total sample size was 144.

## 3. Results

**Figure 1.**
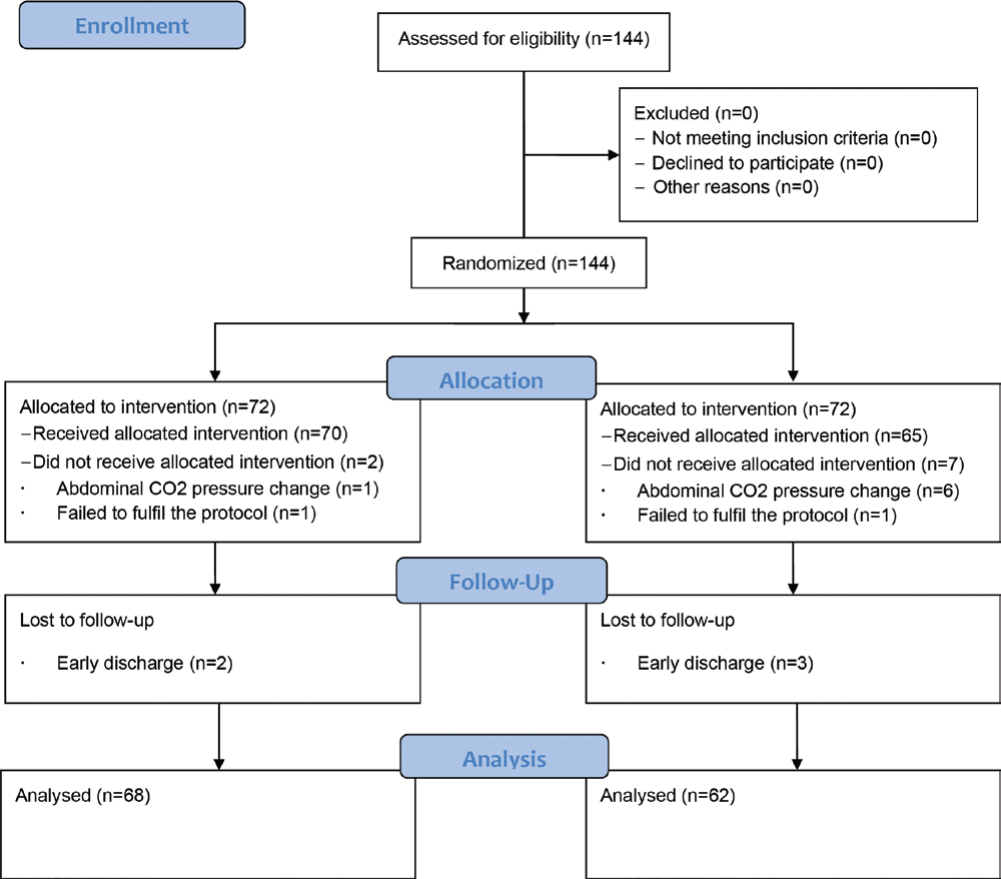
Flow diagram of consolidated standard of reporting trials.

We enrolled 144 patients from September 2021 to May 2022 (Fig. 1). Of the 144 consenting participants, 72 participants were allocated to the 2 groups. Fourteen patients were excluded from this study (7 had abdominal pressure changes due to surgical problems during surgery, 2 did not receive the allocated intervention, and 5 were discharged early and did not sufficiently complete the pain diary). A total of 130 patients were finally analyzed: 68 patients in the AB group and 62 patients in the control group.

**Figure 2.**
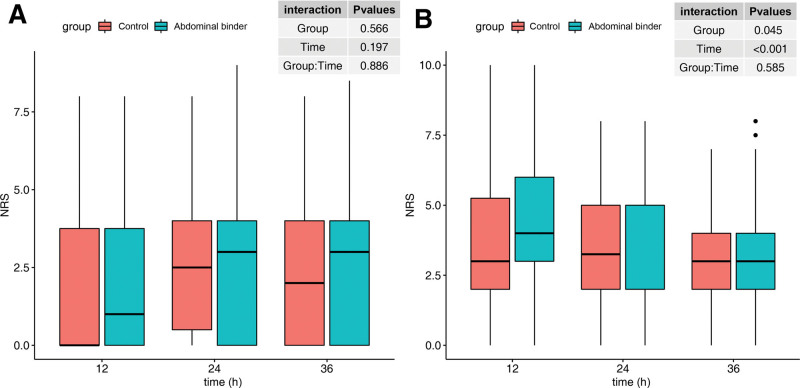
Postoperative pain score at follow-up time points. (A) Shoulder pain. (B) Surgical site pain. Pain scores were measured using the numeric rating scale (NRS). Two-way repeated measure analysis of variance was conducted, and statistical significance set at *P* value < .0167 after Bonferroni correction.

Patient characteristics and intraoperative findings are summarized in Table 1. There were no statistically significant differences between the 2 groups. The incidence and characteristics of postoperative shoulder pain are presented in Table 2. The incidence of postoperative shoulder pain was not different between the 2 groups (58.1% vs 55.9%, risk ratio [95% confidence interval] 0.96 [0.71–1.30], *P* = 1.000). The severity of shoulder pain did not differ between the 2 groups (12 hours: median [interquartile range] 0.0 [0.0–2.0] vs 0.0 [0.0–0.5], *P* = .736; 24 hours: 0.0 [0.0–3.3] vs 0.0 [0.0–3.0], *P* = .737; 36 hours: 0.0 [0.0–3.0] vs 0.0 [0.0–2.0], *P* = .843; worst pain: 2.0 [0.0–5.0] vs 2.0 [0.0–5.0], *P* = .865). Figure 2 shows shoulder pain scores at 3 time points for patients with postoperative shoulder pain. The proportion of positional pain was similar between the 2 groups.

**Table 1:** Patient characteristics.

	Abdominal binder group	Control group	
	(N = 68)	(N = 62)	*P* value
Age, years	43 (36–55)	46 (40–54)	.471
Weight, kg	58.3 (52.4–62.6)	56.7 (51.4–64.4)	.954
Height, cm	160.3 (6.2)	158.9 (6.3)	.182
BMI, kg/m^2^	22.1 (20.5–24.8)	22.9 (20.8–25.1)	.574
ASA-PS classification			.629
1	20 (29.4%)	22 (35.5%)	
2	44 (64.7%)	38 (61.3%)	
3	4 (5.9%)	2 (3.2%)	
Operation name			.118
Biopsy	1 (1.5%)	0 (0.0%)	
Myomectomy	3 (4.4%)	8 (12.9%)	
Ovarian cystectomy	28 (41.2%)	17 (27.4%)	
Salpingo-oophorectomy	19 (27.9%)	14 (22.6%)	
Hysterectomy	17 (25.0%)	23 (37.1%)	
Anesthesia time, h	1.4 (1.1–1.9)	1.6 (1.2–2.1)	.213
Operation time, h	0.9 (0.5–1.3)	0.9 (0.7–1.3)	.297

Data are expressed as numbers (proportions), means (standard deviations), or medians (interquartile ranges).

ASA-PS = American Society of Anesthesiologists Physical Status Classification System, BMI = body mass index.

**Table 2:** Characteristics of postoperative shoulder pain.

	Abdominal binder group	Control group		
	(N = 68)	(N = 62)	Relative risks	*P* value
Shoulder pain, n (%)	38 (55.9%)	36 (58.1%)	0.96 (0.71–1.30)	.941
Positional pain, n (%)*	20/38 (52.6%)	18/36 (50.0%)	1.05 (0.67–1.64)	.822
At postoperative 12 h				
Incidence, n (%)	21 (30.9%)	16 (26.2%)	1.19 (0.69–2.08)	.698
Pain score, NRS	0.0 (0.0–2.0)	0.0 (0.0–0.50)	0 (0–0)	.664
At postoperative 24 h				
Incidence, n (%)	25 (37.3%)	26 (44.1%)	0.88 (0.57–1.34)	.556
Pain score, NRS	0.0 (0.0–3.25)	0.0 (0.0–3.0)	0 (–2 to 0)	.768
At postoperative 36 h				
Incidence, n (%)	24 (35.8%)	20 (33.9%)	1.09 (0.67–1.77)	.969
Pain score, NRS	0.0 (0.0–3.0)	0.0 (0.0–2.0)	0 (0–0)	.796
Worst pain score, NRS	2.0 (0.0–5.0)	2.0 (0.0–5.0)	0 (–2 to 2.5)	.941

Data are expressed as numbers (proportions) or medians (interquartile ranges). Relative risks are represented as the difference in the medians or risk ratio for abdominal binder group relative to control group; differences are (abdominal binder group — control group).

NRS = numerical rating scale.

* : The percentage was calculated only in patients who developed postoperative shoulder pain.

The severity of surgical site pain, pain and nausea medications, and ambulation time did not differ between the 2 groups (Table 3).

**Table 3:** Surgical site pain, rescue medicines, and ambulation time.

	Abdominal binder group	Control group		
	(N = 68)	(N = 62)	Relative risks	*P* value
Surgical site pain				
At postoperative 12 h, NRS	4.0 (3.0–6.0)	3.0 (2.0–5.3)	1 (–1 to 2)	.138
At postoperative 24 h, NRS	4.0 (2.0–5.0)	3.3 (2.0–5.0)	0.75 (–1 to 1.5)	.406
At postoperative 36 h, NRS	3.0 (2.0–4.0)	3.0 (2.0–4.0)	0 (0–1)	.270
Postoperative rescue medicine				
Rescue of pain medicine	18 (26.5%)	14 (22.6%)	1.17 (0.64–2.15)	.756
Rescue of antiemetics	20 (29.4%)	14 (22.6%)	1.30 (0.72–2.35)	.493
Ambulation time, h	21.0 (19.0–23.0)	21.0 (18.0–23.5)	0 (–1.83 to 2)	.588

Data are expressed as numbers (proportions) or medians (interquartile ranges). Relative risks are represented as the difference in the medians or risk ratio for abdominal binder group relative to control group; differences are (abdominal binder group — control group).

NRS = numerical rating scale.

## 4. Discussion

In this study, AB use did not provide any additional benefit compared to the use of the pulmonary alveolar recruitment maneuver alone following laparoscopic gynecologic surgeries. A previous study on the efficacy of AB on postoperative pain control showed that the association between time and postoperative shoulder pain differed according to AB status.^[8]^ This study was the first to compare the AB status by frequency of shoulder pain as a primary outcome measure after laparoscopic gynecologic surgery. We expected that the AB would reduce postoperative shoulder pain; however, our study showed that using an AB did not reduce postoperative shoulder or surgical site pain following laparoscopic gynecologic surgery.

CO_2_ is commonly used for laparoscopic surgery because of its high solubility in blood and low reactivity. However, CO_2_ could remain after pneumoperitoneum desufflation. The residual CO_2_ adjacent to the diaphragm can irritate the end of the phrenic nerve. Excessive stretching of the diaphragm due to pneumoperitoneum pressure is another explanation for postoperative shoulder pain. Pressure on the phrenic nerve, which originates from the C3 to C8 roots and ends on the diaphragm, may cause shoulder pain.^[8,9]^

Various interventions have been proposed to ameliorate postoperative shoulder pain. To reduce the remaining CO_2_, a pulmonary alveolar recruitment maneuver^[3]^ filling the abdominal cavity with normal saline^[10]^ and active evacuation of CO_2_^[11]^ have been introduced to decrease postoperative shoulder pain. To prevent excessive stretching of the diaphragm, which causes postoperative shoulder pain, the following strategies were proposed: low CO_2_ flow rate,^[12]^ low intraabdominal pressure,^[13]^ and deep muscular block.^[14]^ Nevertheless, the best technique for resolving shoulder pain has not been identified.

Recently, a study reported that the postoperative Trendelenburg position decreased the frequency and severity of postoperative shoulder pain after gynecologic laparoscopic surgery.^[15]^ The Trendelenburg position aided residual CO_2_ migration to the pelvis, which has an ampule vasculature and promoted residual CO_2_ resorption. However, forcing patients to maintain the Trendelenburg position for 24 hours could be challenging. Hence, we attempted to induce CO_2_ movement in this study by performing the pulmonary recruitment maneuver in the Trendelenburg position and increasing abdominal pressure using an AB to facilitate CO_2_ absorption. We expected an increase in intraabdominal pressure when the AB was worn. In addition, wearing the AB is easy and inexpensive.

Despite the use of ABs, the frequency and severity of postoperative shoulder pain did not decrease in this study. There are possible explanations for this ineffectiveness. First, the increase in abdominal pressure from the AB was insufficient to promote residual CO_2_ absorption. In a healthy adult, the normal intraabdominal pressure ranges from 5 to 7 mm Hg.^[16]^ It is reported that the abdominal pressure increases by 1.1 ± 0.7 mm Hg when using an elastic binder.^[17]^ The CO_2_ solubility coefficient was 0.0308 mmol/L/mm Hg.^[18]^ When wearing an AB, the intraabdominal pressure increases by approximately 1 mm Hg, resulting in a further dissolved amount of CO_2_ of 0.0308 mmol/L. This may not be sufficient to prevent postoperative shoulder pain. Second, the AB may form a compartment, preventing CO_2_ adjacent to the diaphragm from reaching the pelvis. Free air in radiologic images is used to identify the location of perforation in the acute abdomen.^[19]^ This might indicate that free air does not move easily. To prevent trapping CO_2_ near the diaphragm, we conducted sequential procedures for the patients after surgery. First, the pulmonary recruitment maneuver in the Trendelenburg position facilitates the caudal movement of CO_2_. Following this, we applied an AB while maintaining the same position. After these procedures, the patient’s position was changed to supine. Our intention was to minimize the accumulation of CO_2_ adjacent to the diaphragm. However, removal of CO_2_ may not have been insufficient, and the AB might aggravate isolation of remaining CO_2_. This result indirectly suggests that CO_2_-induced phrenic nerve irritation is a contributing factor to shoulder pain after laparoscopy.

The impact of using an AB after laparoscopic surgery on pain and recovery remains controversial. A previous study in gynecologic surgery showed that using an AB did not improve postoperative pain or respiratory function^[20]^; however, another study showed reduced postoperative pain with an AB in the first week.^[21]^ In a study of patients undergoing incisional hernia repair surgery, AB use reduced postoperative pain,^[22]^ but similar findings were not observed in patients undergoing umbilical or epigastric hernia repair surgery.^[23]^ In our study, AB use did not reduce postoperative surgical site pain or ambulation time. However, because our protocol did not use the enhanced recovery after surgery system, early ambulation was not enforced. Overall, the advantages of using an AB may be insignificant, and further studies are required.

### 4.1. Limitations

There were several limitations in this study. First, the follow-up period was 36 hours. This time frame was chosen because most patients undergoing laparoscopic gynecologic surgery at this institute were discharged on the morning of the second day after surgery. However, 7 patients first complained of shoulder pain at postoperative 36 hours. A longer follow-up period may be required for understanding the characteristics of shoulder pain. Second, owing to the nature of ABs, double blinding was impossible. The patients knew if they would or would not be wearing an AB while signing the consent form. This could influence the investigation of pain. Third, it was difficult to guarantee that the patient would continue to wear an AB, although the patient was asked to wear it continuously. The AB could be removed for some time such as to check dressings or the drainage bag. Third, 12L/min is a relatively high insufflation flow which was reported to associate with postoperative shoulder pain.^[12]^ Although the insufflation flow rate is unified in 2 groups, it should be interpreted with caution, as it can lead to different results at lower flows.

### 4.2. Conclusions

The use of an AB was not beneficial in terms of postoperative shoulder pain in laparoscopic gynecologic surgery. Moreover, surgical site pain, ambulation time, and postoperative nausea and vomiting did not improve with the use of an AB. Based on our results, AB use is not recommended in patients undergoing laparoscopic gynecologic surgery.

## Author contributions

**Conceptualization:** Hee-Soo Kim.

**Data curation:** Yoon Jung Kim, So Yeong Hwang.

**Formal analysis:** Yoon Jung Kim.

**Investigation:** Yoon Jung Kim.

**Supervision:** Hee-Soo Kim.

**Writing – original draft:** Yoon Jung Kim.

**Writing – review & editing:** Yoon Jung Kim, Hee-Soo Kim, So Yeong Hwang.
